# Brain controllability mediates the effects of early life adversity on adolescent behavior and cognition moderated by genetic risks

**DOI:** 10.21203/rs.3.rs-8777490/v1

**Published:** 2026-03-19

**Authors:** Huaigui Liu, Yurong Jiang, Huili Sun, Matthew Rosenblatt, Jean Ye, Chunshui Yu, Dustin Scheinost

**Affiliations:** Yale university; Yale University; Yale University; Yale University, New Haven, Connecticut, USA; Tianjin Medical University General Hospital; Yale School of Medicine

## Abstract

Early life adversity (ELA) is a robust transdiagnostic risk factor for mental health disorders, yet the neurobiological mechanisms mediating its long-term impact remain poorly understood. Network control theory offers a novel framework for capturing how structural brain networks constrain and support brain dynamics. Controllability increases over development, associates with executive function and mental health, and appears sensitive to environmental insults. Thus, it may reflect a neurobiological mediator between ELA and behavioral outcomes. We tested whether alterations in modal controllability mediate the impact of multidimensional ELA on cognitive and behavioral outcomes in youth, and whether these pathways are shaped by genetic risk for neurodevelopmental conditions. Using data from 7,970 children aged 9–11 years in the Adolescent Brain Cognitive Development (ABCD) Study, we derived five latent ELA dimensions from 67 indicators, and computed polygenic risk scores (PRS) for attention-deficit/hyperactivity disorder (ADHD_PRS_) and autism spectrum disorder (ASD_PRS_). Distinct ELA dimensions were associated with increased controllability in medial frontal, frontoparietal, default mode, and motor networks, as well as with externalizing symptoms and impaired crystallized cognition. Controllability partially mediated these associations, and indirect effects were significantly moderated ADHD_PRS_ and ASD_PRS_. Longitudinal analyses further demonstrated that baseline controllability predicted cognitive performance two years later. These findings delineate a neurodevelopmental cascade linking early adversity and genetic vulnerability to transdiagnostic mental health risk, positioning brain controllability as a promising mechanistic marker and potential target for early intervention.

## Introduction

Early life adversity (ELA) is a potent and pervasive determinant of emotional, cognitive, and behavioral development. Mounting evidence links it with elevated risk for psychiatric disorders across the lifespan [1–6]. However, as adversity includes a wide-range of factors (e.g., caregiver psychopathology, emotional maltreatment, family dysfunction, socioeconomic disadvantage, neighborhood crime and trauma [7]), it is not a unitary construct. Furthermore, these distinct dimensions of ELA may exert differential effects on developmental trajectories [8, 9]. For instance, deprivation is linked to executive function deficits and significantly higher rates of ADHD [10, 11], unpredictability is shown to correlate with more conduct problems [12], while physical abuse is associated with increased lifetime substance use [13]. These distinctions motivate multidimensional models of ELA to better capture its heterogeneity and co-occurrence. These models offer a more precise framework for studying developmental consequences of ELA.

The brain is particularly vulnerable to the effects of ELA [14]. Structural and functional neuroimaging studies have consistently demonstrated that ELA disrupts neurodevelopmental trajectories in domain-specific and region-specific ways [15, 16]. For example, deprivation-related adversity are associated with reduced total brain volume [17–19] and premature adult-like connectivity between the amygdala and the medial prefrontal cortex [20]. While maltreatment exhibit cortical thinning in prefrontal and temporal regions [21–23] and heightened amygdala connectivity with the salience, default mode, and prefrontal networks [24]. White matter abnormalities have also been reported across distinct types of adversity: frontostriatal alterations following early institutionalization, neglect and deprivation [25–27], frontolimbic disruptions linked to socioeconomic disadvantage and physical neglect [28, 29], and reduced integrity of association fibers in individuals exposed to domestic violence [30]. Yet, despite this growing literature, a fundamental question remains: how do adversity-related alterations in brain networks translate into increased vulnerability for psychopathology?

One promising avenue for addressing this question lies in network control theory, a computational framework grounded in systems neuroscience [31]. Rather than describing regional structure or function in isolation, network controllability quantifies the brain’s capacity to flexibly transition between diverse mental states, based on the underlying structural connections [31]. Thus, network control theory offers a biologically grounded and mathematically tractable marker of structural connections constrain and support brain dynamics. Controllability increases over young childhood through adolescence, putatively allowing for the emergence of adult-like executive functions [32, 33]. Given that executive functions are both developmentally sensitive and highly vulnerable to adversity [34, 35], controllability may serve as a critical link between structural network changes and behavioral outcomes. Further, controllability is altered in several psychiatric disorders [36–38]. However, its role in adversity-psychopathology pathways remains largely unexplored.

Importantly, not all children exposed to ELA develop psychiatric disorders. Genetics may explain this variability [39, 40]. Previous studies estimate that up to 40–80% of the variance in psychiatric disorders is heritable [41], and polygenic risk scores (PRS) derived from genome-wide association studies (GWAS) now offer a scalable approach to quantify individual-level genetic liability [42]. Prior studies have linked PRSs of psychiatric disorders to brain structure [43], but few have examined their interaction with ELA in mediating neurodevelopmental pathways. Understanding these interactions is essential, especially in youth, when both environmental exposures and brain networks remain plastic.

In the present study, we leveraged data from the ABCD study to investigate how distinct dimensions of ELA affect brain network controllability, and how these changes may mediate cognitive and behavioral outcomes. We also tested whether genetic risk for ADHD and ASD moderates these brain behavior pathways, and whether the observed effects persist over a two-year longitudinal follow-up. We selected risk for ADHD and ASD as they are early-onset neurodevelopmental conditions with high heritability and relevance to the age range of the ABCD cohort. Additionally, both are associated with altered structural connections [44–47]. To this end, we applied dimensional modeling of ELA across 67 baseline indicators, computed controllability metrics across ten conical brain networks, and assessed behavioral and cognitive outcomes using the Child Behavior Checklist (CBCL) and NIH Toolbox at both baseline and follow-up. Analyses included linear mixed-effects models, mediation and moderated mediation frameworks, and cross-lagged panel model (CLPM) cross-lagged panel modeling to examine temporal dynamics. By integrating environmental, neural, behavioral, and genetic data in a large, diverse pediatric sample, this study aims to elucidate the mechanisms through which early adversity becomes biologically embedded, and to identify potential targets for early identification and intervention in psychiatric risk.

## Methods

### Participants

All participants were recruited by the ABCD study from the ABCD consortium (https://abcdstudy.org/index.html), which includes over 11,000 children aged 9–11 years, recruited from 22 sites across the United States with a diverse range of geographic, socioeconomic, ethnic, and health backgrounds [48]. We used the Annual Curated Data Release 5.1. The study protocols were approved by the Institutional Review Boards (IRBs) at each participating site, with written informed consent obtained from youth and caregivers. Details about the participant cohort, data collection procedures, and preprocessing protocols are provided at the ABCD website (https://abcdstudy.org/scientists/protocols/) and also are described elsewhere [48, 49].

### Early Life Adversity

#### Item Selection and Data Cleaning

ELA was assessed at baseline using 139 variables selected from the ABCD baseline measures [7, 50], which captured diverse adversity domains, including caregiving disruption, caregiver psychopathology, maltreatment, neighborhood violence, family and community support, socioeconomic disadvantage, and exposure to physical trauma (**Supplementary Table 1**). All items were drawn from validated scales administered to the child, parent, or rated by trained research staff and were coded such that higher scores indicated greater adversity.

We excluded 31 variables with > 50% missing and removed an additional 29 variables with zero or near-zero variance using the *nearZeroVar* function from the caret R package [51]. Two redundant variables were manually removed. Furthermore, eight participants lacking age information were excluded. To avoid multicollinearity arising from familial relatedness, we excluded twins and triplets, retaining at most one participant per family among non-twin siblings. This exclusion was applied prior to all analyses, resulting in a final analytic sample of N = 2,054 excluded participants. After these data cleaning procedures, the remaining variables underwent outlier detection, with outliers defined as values exceeding 4 × median absolute deviation (MAD) of continuous environmental variables [52]. Finally, 10 ELAs variables with a missing rate > 50% were excluded and a final set of 67 variables was included. Details are provided in **Supplementary Fig. 1, 2** and **Supplementary Table 1**. Missing values were handled using the *mice* (v3.16.0) [53] R package. Ten imputed datasets were generated using predictive mean matching (continuous variables) and logistic regression (categorical variables), with 10 iterations each.

#### Factor Analysis and Validation

To reduce dimensionality and identify core latent adversity factors, we conducted an exploratory factor analysis (EFA) using principal axis factoring and *oblimin* rotation. ELA variables were averaged across the 10 imputed datasets prior to factor analysis. Suitability for factor analysis was confirmed by Kaiser-Meyer-Olkin (KMO) values > 0.60 and significant Bartlett’s test of sphericity (p < .001). The number of factors was determined by a combination of Very Simple Structure (VSS) and Minimum Average Partial (MAP) criteria, supplemented by model fit indices, including comparative fit index (CFI), root mean square error of approximation (RMSEA) and standardized root mean square residual (SRMR) across 1–9 factor solutions.

Indicators with loadings > 0.45 were retained as primary contributors to each factor. Final factor structures were validated through confirmatory factor analysis (CFA) using the *lavaan* R package (version 0.6–19). To reduce overfitting, we applied tenfold cross-validation: in each fold, 90% of participants were used to estimate factor loadings, while factor scores were computed in the remaining 10%. This was iterated until all participants had out-of-sample estimates. The stability of factor scores was confirmed using Spearman correlations between cross-validated and full-sample scores.

### Behavioral Measures

#### Child Behavior Checklist (CBCL)

Children’s emotional and behavioral symptoms were assessed using the Child Behavior Checklist (CBCL), a validated parent- or caregiver-report scale [54]. The CBCL includes eight syndrome scales: anxious/depressed, withdrawn/depressed, somatic complaints, social problems, thought problems, attention problems, rule-breaking behavior, and aggressive behavior. We used standardized T-scores of three composite subscales: internalizing problems (anxious/depressed, withdrawn/depressed, and somatic complaints), externalizing problems (rule-breaking and aggressive behaviors), and total problems (all eight syndrome scales combined), with higher scores indicating greater symptom severity.

#### NIH Toolbox Cognition Battery

Cognitive function was evaluated using the NIH Toolbox Cognition Battery [55], which consists of seven performance-based tasks designed to measure key cognitive domains. Crystallized intelligence was assessed by summing performance on the Picture Vocabulary Test and the Oral Reading Recognition Test. Fluid intelligence was calculated using five tasks: the Flanker Inhibitory Control and Attention Test, the List Sorting Working Memory Test, the Dimensional Change Card Sort Test, the Pattern Comparison Processing Speed Test, and the Picture Sequence Memory Test. A total cognitive composite score was derived by summing the fluid and crystallized scores. For all cognition domains, standardized T-scores were used in the analyses, with higher values reflecting better cognitive performance. Crystallized intelligence data were obtained at both the baseline and 2-year follow-up.

#### Neuroimaging Data Acquisition and Processing

Diffusion MRI (dMRI) data were acquired using standardized protocols across multiple 3T scanner platforms at ABCD sites [48]. Individual-level diffusion MRI data were downloaded in FIB format from the ABCD Study Data Portal (https://brain.labsolver.org), where the preprocessing pipeline is detailed (N = 9,713 for baseline, N = 7,197 for 2-year follow-up). Raw dMRI volumes were acquired using a multishell diffusion scheme with b-values of 500, 1000, 2000, and 3000 s/mm^2^ and corresponding diffusion directions of 6, 15, 15, and 60, respectively. Images were acquired at an in-plane resolution of 1.7 mm and slice thickness of 1.7 mm, and were rotated to align with the anterior commissure–posterior commissure (AC–PC) line, resulting in isotropic voxel resolution of 1.7 mm.

Diffusion data were reconstructed using generalized q-sampling imaging with a sampling length ratio of 1.25 [33, 56, 57]. Whole-brain tractography was performed in DSI Studio using deterministic streamline tracking with a modified FACT algorithm [57]. Tractography parameters included an angular cutoff of 60 degrees, step size of 1.0 mm, minimum streamline length of 30 mm, maximum length of 300 mm, and spin density function smoothing set to 0. QA values were used as the termination criterion, and fiber tracking continued until 10 million streamlines were reconstructed for each subject.

QA was computed in native space and used for nonlinear registration to MNI space via the SPM12 algorithm. Within MNI space, spin density functions were reconstructed using three fiber orientations per voxel, and a mean diffusion distance of 1.25 mm. For each participant, a 268 × 268 structural connectivity matrix was generated by averaging the QA values of streamlines connecting pairs of regions from the 268-node Shen atlas [58]. Only connections with mean QA > 0.001 were retained to minimize noise. These 268 parcellated regions were grouped into 10 canonical networks [59]: medial frontal (MF), frontoparietal (FP), default mode (DMN), motor (Mot), visual I (Vis I), visual II (Vis II), visual association (Vas), salience (SAL), subcortical (SC), and cerebellar (CBL) networks.

### Network Control Theory and Controllability Metrics

#### Dynamical Model

The network control theory can simulate the consequences of a network’s underlying structural topology on its function dynamics [60]. The present study applied the network control theory framework to calculate brain controllability [31–33], implemented in MATLAB with in-house packages and code, from structural connectomes. Based on the previous studies [31, 32], neural states can be mathematically described as simulated states (x) of network with k nodes overtime steps t using a simplified noise-free linear discrete-time and time-invariant model:

$$x(\backslash varvect+1)=Ax(\backslash varvect)+B_{k}u_{k}(\backslash varvect)$$

Where \varvecx(\varvect) is a vector that describes the state of all brain regions at time t, while matrix A is a structural connection matrix. K is the set of nodes that can be controlled independently. The input matrix defines the control nodes. The $u_{K}\;(\backslash varvect)$ is the control signal injected into the network via the control nodes, while B is the input matrix.

#### Controllability Metrics

Subsequently, the influence of each region on brain function is quantified using the metric of controllability. Controllability of a dynamical system refers to the possibility of driving the state of a dynamical system to a specific target state by means of an external control input. Classic results in the control theory ensure that controllability of the network from the set of network nodes k is equivalent to the controllability Gramian $W_{K}$ being invertible, where

$$varvecW_{\backslash varveck}=\sum_{\backslash varvec\tau =0}^{\backslash arvec\infty }varvecA^{\backslash varvec\pi }\backslash varvecB_{\backslash varveck}\backslash varvecB_{\backslash varveck}^{\backslash \mathrm{va}rvecT}\backslash varvecA^{\backslash varvec\pi }$$

We utilize this framework to choose control nodes one at a time, and thus the input matrix B in fact reduces to a one-dimensional vector.

Based on this network control theory framework, we examined modal controllability, which quantifies the ability of a brain region to drive the network into difficult-to-reach states that require substantial control energy [31]. Such transitions may correspond to cognitively demanding or less frequently engaged brain states [32], which are particularly relevant in the context of neurodevelopment and psychopathology. Modal controllability is computed from the eigenvectors $\left[\backslash varvec\;V_{\backslash varveci\backslash varvecj}\right]$ of the adjacency matrix A. Here, we define the measurement of modal controllability as $\backslash varvec\Phi_{\backslash varveci}=\sum_{\backslash varvecj=1}^{\backslash varvecN}\left(1-\backslash varvec\lambda_{\backslash varvecj}^{2}(\backslash varvecA)\backslash varvecV_{\backslash varveci\backslash varvecj}^{2}\right)\text{of}\;\text{all}\;\text{N}$ of all N modes $\backslash varvec\lambda_{0}(\backslash varvecA),\ldots ,\backslash varvec\lambda_{N-1}(\backslash varvecA)$ from brain region i, following the definition of previous studies.

### PRSs Calculation

#### Base GWAS Datasets

PRS_ADHD_ and PRS_ASD_ were derived for two childhood psychiatric conditions: attention deficit hyperactivity disorder (ADHD) [61], and autism spectrum disorder (ASD) [62], using GWAS summary statistics from cohorts of European ancestry. These disorders were selected based on their well-powered GWAS, relevance to early development, and broad clinical presentations. The base datasets included single nucleotide polymorphisms (SNPs) with reported effect alleles, odds ratios (ORs), P-values, and allele frequencies. Full details of the GWAS samples and cohort sizes are provided in **Supplementary Table 2**.

#### Target Genotype Dataset and Quality Control

Due to the reduced performance of cross-ancestry prediction [63, 64], analyses were restricted to a subset of participants of confirmed European ancestry. Genotype data were obtained from the ABCD Study (Release 5.1) and processed using Axiom Analysis Suite (version 2.11) from raw intensities files generated by the Affymetrix Smokescreen array. Biological samples were derived from either saliva or whole blood samples. Participants were selected based on genotyping call rate, concordance between reported and genetic sex, and identity-by-state (IBS) estimates to minimize cryptic relatedness. Initial quality control (QC) was conducted by the ABCD consortium and included call rate thresholds, signal intensity checks, and marker-level filters, followed by the RICOPILI pipeline for imputation-ready QC.

Ancestry-specific quality control was then applied separately to participants of European ancestry and to the remaining trans-ancestry group. SNPs were filtered based on missingness rate (< 5%), Hardy-Weinberg equilibrium (HWE < 1×10^−9^), and excess heterozygosity (> 3 SD from the mean). Genotype phasing and imputation were performed using the TOPMed imputation server. Post-imputation SNPs were retained only if they had a minor allele frequency (MAF) ≥ 0.005 and imputation quality INFO score ≥ 0.9. A pruned set of SNPs (clumping threshold *r*^*2*^ = 0.1) was used to calculate the top 10 genetic principal components (PCs) using PLINK [65]. Due to the reduced performance of cross-ancestry prediction [63, 64], all PRS analyses were restricted to a subset of participants of confirmed European ancestry.

### PRS Computation

PRS were computed using PRSice-2 (version 2.3.5) [66] with clumping and thresholding parameters set as clump-kb 250kb, clump-r^2^ 0.1, and clump-p 1.0. For each participant, PRS were calculated as the sum of risk alleles (0, 1, or 2) at each locus, weighted by the corresponding log-transformed OR (logOR) from the base GWAS. Resulting scores were standardized (z-scored) prior to inclusion in downstream analyses.

### Covariates

To reduce potential confounding, all analyses were adjusted for a consistent set of covariates, including age, sex, race, study site, handedness, pubertal status [67], and the first 5 genetic principal components. Categorical variables (sex, race, handedness) were dummy-coded prior to inclusion in statistical models.

### Statistical Analysis

All continuous variables were standardized (Z-score) prior to analysis to account for differences in measurement scales. The Benjamini-Hochberg false discovery rate (FDR) correction was applied to adjust for multiple comparisons, and adjusted *q*-values were reported [68].

### Association Analysis at Baseline

The linear mixed-effects models were employed to examine the pairwise associations among ELA latent factors, controllability measures, and behavioral assessments at baseline. In each model, controllability or behavioral measure was used as the dependent variable, ELA factor and all covariates as fixed effects, and the study site as a random effect [69]. The estimated regression coefficient (β), standard error and *p* value were reported. Additionally, we estimated pairwise Pearson correlation between controllability and behavioral measures.

### Stratified Analysis

To evaluate the robustness and generalizability of the observed association, sex-stratified and ancestry-stratified analyses were performed. Models were re-estimated separately for males and females, and again for participants of European versus non-European ancestry. To formally assess the similarity of results across subgroups, we calculated Pearson correlation between stratified effect sizes across all brain networks or behavioral outcomes. When low cross-group consistency was observed, additional interaction analyses between sex or ancestry group and each key independent variable were conducted in the full sample models. These interaction models allowed us to directly assess whether the strength or direction of associations differed significantly across sex or ancestry groups.

### Mediation and Moderated Mediation Analyses

When brain controllability was significantly associated with both ELA latent factors and behavioral measures, mediation analyses were conducted to test whether the controllability measure mediated the associations between ELA factors and behavioral measures. Indirect effects were estimated using a bias-corrected bootstrap procedure with 5000 iterations, and mediation was considered statistically significant if the 95% CI did not include zero.

For each significant mediation pathway, moderated mediation analyses were then conducted in the European subsample to examine whether the indirect effect varied by genetic risks for ADHD and ASD. Participants were divided into low (15%), medium (70%), and high (15%) genetic risk subgroups based on the 16th, and 84th percentiles, consistent with the default settings in the *PROCESS macro* for R [70]. Conditional indirect effects were estimated for each group using the same bootstrap approach.

### Longitudinal Analysis

A cross-lagged panel model (CLPM), implemented with the *lavaan* package in R [71], was applied to evaluate associations between baseline brain controllability measures and 2-year follow-up behavioral outcomes, and vice versa. These analyses were performed in participants with follow-up behavioral assessments (5,115 for CBCL and 3,999 for crystallized intelligence). Prior to model fitting, both brain controllability metrics and behavioral scores (CBCL scales and cognition) at baseline and 2-year follow-up were residualized by regressing out all covariates using linear models. The resulting residuals were then standardized (z-scored) to ensure comparability across variables. Cross-lagged associations were estimated between baseline and follow-up measures of brain and behavior. Each CLPM included autoregressive paths (baseline to follow-up) and cross-lagged paths (brain to behavior and vice versa), with correlated residuals specified at both timepoints. All models were estimated using the *sem* function from the *lavaan* R package with full-information maximum likelihood (ML) estimation and robust (Huber-White) standard errors. To control for multiple testing, false discovery rate (FDR) correction was applied across all *p*-values derived from cross-lagged paths.

## Results

### Demographics

A total of 7,970 participants were included (**Supplementary Fig. 1**), comprising 4,141 males (52.0%) and 3,829 females (48.0%). The mean age of the participant was 9.47 years at baseline (standard deviation [SD] = 0.51; range = 9–11 years). Participants were identified as Asian (1.7%), Black (15.0%), Hispanic (20.1%), White (53.0%), and Other Race (10.2%). Detailed demographic characteristics of the participants are summarized in Table 1.

### ELA Dimensions

To derive latent dimensions of early life adversity (ELA), we first applied quality control to 139 candidate variables (**Supplementary Fig. 1 and** Table 1), retaining 67 indicators for exploratory factor analysis (EFA) (**Supplementary Fig. 2**). The data were suitable for factor analysis, as evidenced by Kaiser-Meyer-Olkin statistic = 0.827 and Bartlett test of sphericity (χ^2^ = 266 144, *p* < .001). To determine the optimal number of latent factors, we used Very Simple Structure (VSS), Minimum Average Partial (MAP), and model fit indices. VSS suggested four and five factors as optimal solutions under complexity levels 1 and 2 (VSS = 0.553 and 0.614, respectively). Although the MAP statistic reached its global minimum at nine factors (MAP = 0.0048), values were near-minimal at four (MAP = 0.0058) and five (MAP = 0.0054) factors, indicating that these solutions captured the most common variance. Moreover, root mean square error of approximation (RMSEA) decreased progressively and plateaued at five factors (RMSEA = 0.027), supporting the selection of a five-factor model (**Supplementary Fig. 3**). Confirmatory factor analysis (CFA) of the five-factor solution showed excellent model fit in 10-fold cross-validation (**Supplementary Table 3**), with cross-validated factor scores highly correlated with full-sample scores (Spearman *ρ* range, 0.99–1.00), supporting the stability and robustness of the five-factor model.

**Figure 1:**
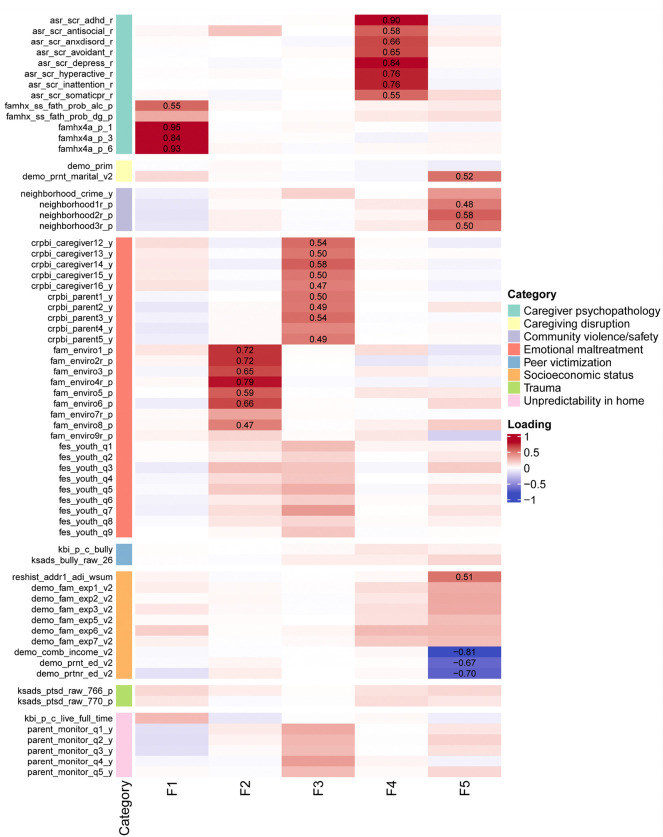
Standardized Factor Loadings of Early Life Adversities for the 5-Factor Solution. Heatmap showing standardized loadings (≥|0.45| labeled) of 67 adversity indicators on five latent factors (F1-F5). Higher factor scores represent greater exposure to adversity. Color scale indicates loading magnitude and direction (red=positive, blue=negative). Categories of ELA are color-coded on the left margin. Abbreviations: F1: Paternal psychosocial adversity, F2: Family conflict/hostile environment, F3: Low caregiver and emotional support, F4: Caregiver psychopathology, F5: Socioeconomic disadvantages.

The five latent ELA dimensions represented distinct psychosocial contexts (Fig. 1, **Supplementary Table 4**). Factor 1 (F1) reflected paternal psychosocial adversity, including high loadings on father-related marital, occupational, legal, behavioral, and substance use problems. Factor 2 (F2) captured family conflict and hostile climate, characterized by frequent fights, criticism, physical aggression, and low conflict resolution. Factor 3 (F3) indicated low caregiver and emotional support, with high loadings on less frequent caregiver comfort, love expression, open communication, and emotional availability. Factor 4 (F4) represented caregiver psychopathology, with high loadings on caregiver depressive, anxiety, somatic, ADHD, antisocial, and avoidant symptoms. Factor 5 (F5) indexed socioeconomic disadvantages, with high loadings for family income (negative loading), parental education (negative loading), and neighborhood safety concerns (positive loading). Together, these dimensions offer a comprehensive, multidimensional characterization of ELA during early development.

### Associations between ELA and Brain Controllability

**Figure 2:**
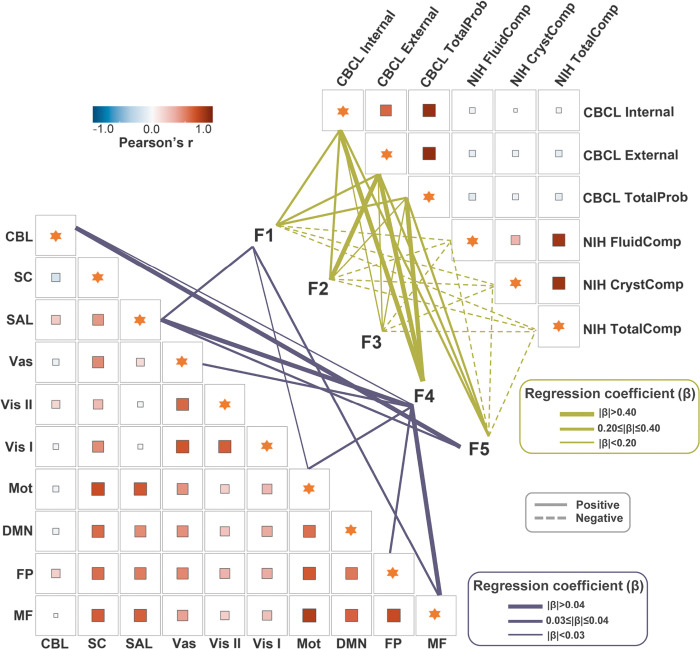
Associations of ELA Factors with Brain Controllability and Behavioral outcomes. Solid lines represent significant positive associations (*q* < .05) between ELA factors (F1-F5) and brain controllability across 10 canonical networks, with line thickness indicating regression coefficients. Dashed lines denote significant negative associations. Similarly, associations between ELA factors and behavioral/cognitive outcomes are shown on the right, with line thickness reflecting regression coefficients. Color shading in the lower-left triangle represents Pearson’s correlation coefficients (r) between brain controllability and behavioral outcomes. These correlation results are supplementary and provided for reference only (see **Supplementary Fig.4, 5**). Abbreviations: CBL, cerebellar network, DMN, default mode network, F1: Paternal psychosocial adversity, F2: Family conflict/hostile environment, F3: Low caregiver and emotional support, F4: Caregiver psychopathology, F5: Socioeconomic disadvantages, FP, frontoparietal network, MF, medial frontal network, Mot, motor network, SAL, salience network, SC, subcortical network, Vas, visual association network, Vis I, visual network I, Vis II, visual network II.

Three ELA dimensions were significantly associated with brain controllability (Fig. 2, **Supplementary Table 5**). We identified consistent positive correlations between F1 (paternal psychosocial adversity) and controllability in the medial frontal, motor, and salience networks (*β* = 0.028–0.036; *q* < .05), F4 (caregiver psychopathology) and controllability in the salience, medial frontal, visual association, motor, frontoparietal, and cerebellar networks (*β* = 0.030–0.047; *q* < .05), and F5 (socioeconomic disadvantage) and controllability in the cerebellar and salience networks (*β* = 0.039–0.057; *q* < .05). These findings indicate that distinct ELA dimensions could consistently increase brain controllability, with both shared and unique effects. However, F2 and F3 were not significantly associated with controllability in any network.

### Associations between ELA and Behavioral Outcomes

All five ELA factors were significantly associated with behavioral outcomes (Fig. 2, **Supplementary Table 6**). Higher ELA factor scores, especially F4 scores, were consistently associated with greater internalizing, externalizing, and total behavioral problems on the CBCL (*β* = 0.091 to 0.564; *q* < .05). All, but F4, were associated with lower cognition composite scores (*β* = −0.029 to −0.167; *q* < .05), particularly for F5 (*β* = −0.120 to −0.167; *q* < .05).

### Association between Controllability and Behavioral Outcomes

**Figure 3:**
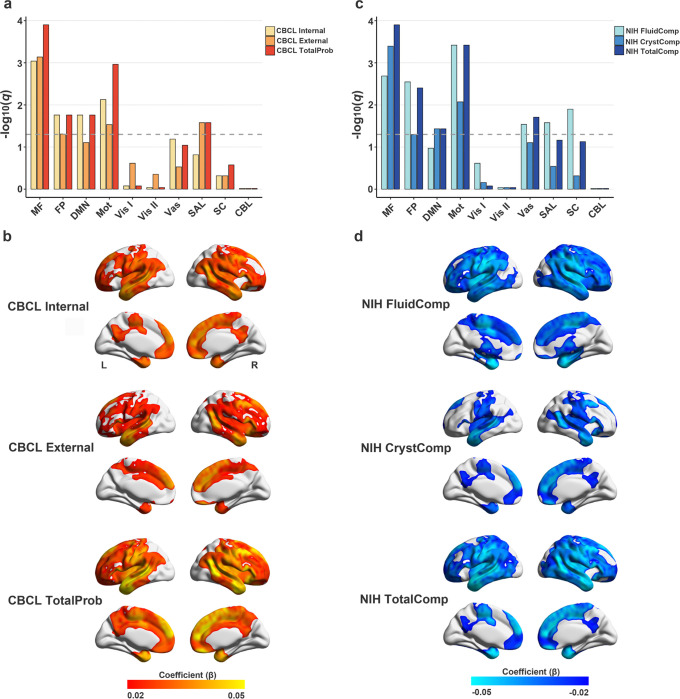
Associations Between Brain Controllability and Behavioral Outcomes. Associations of brain controllability with behavioral symptoms (A) and cognitive measures (C). Bars represent the −log10(*q*) of FDR-corrected *p*-values across ten canonical brain networks. The dashed line indicates the FDR significance threshold (*q* = .05). (B, D) Cortical surface maps of standardized regression coefficients (*β*) for associations between network-level brain controllability and behavioral measures. Warmer colors indicate stronger positive associations, cooler colors indicate stronger negative associations. Abbreviations: CBL, cerebellar network, DMN, default mode network, FP, frontoparietal network, MF, medial frontal network, Mot, motor network, SAL, salience network, SC, subcortical network, Vas, visual association network, Vis I, visual network I, Vis II, visual network II.

Higher controllability in the medial frontal, frontoparietal, default mode, motor, and salience networks was significantly associated with greater behavioral problems (*β* = 0.031 to 0.052; *q* < .05, Fig. 3A/B, **Supplementary Table 7**). In particular, Higher medial frontal and motor network controllability was consistently associated with higher externalizing (MF: *β* = 0.044; Mot: *β* = 0.028) and internalizing (MF: *β* = 0.043; Mot: *β* = 0.036) symptoms (all *q* < .05). However, SAL network controllability was also correlated with externalizing symptoms (*β* = 0.030; *q* < .05), while FP and DMN network controllability was additionally associated with internalizing symptoms (FP: *β* = 0.031; DMN: *β* = 0.033; *q* < .05).

Worse cognitive performance was also linked to higher controllability across seven networks, including the medial frontal, frontoparietal, default mode, motor, salience, visual association, and subcortical networks (Fig. 3C/D, **Supplementary Table 8**). Specifically, higher controllability in the medial frontal, default mode, and motor networks was linked to lower crystallized cognition composite scores (*β* = −0.027 to −0.047; *q* < 0.05).

### Stratified Analyses

In sex-stratified analyses, ELA-brain controllability and brain controllability-behavior associations were generally consistent across males and females (*r* = 0.552 to 0.993, *q* < .05) (**Supplementary Fig. 6**). However, there were several potential sex-specific effects. For ELA, F4 and F5 showed stronger associations with controllability in the cerebellar network for females (**Supplementary Fig. 7**). F5 showed stronger associations with CBCL total problems in males (*β* = 0.282 vs 0.241 in females, **Supplementary Fig. 8**). Controllability in the subcortical, frontoparietal, and medial frontal networks were more strongly associated with cognitive total composite score in females (**Supplementary Fig. 9**).

In contrast, ancestry-stratified analyses revealed divergence in specific domains (**Supplementary Fig. 10**). Associations between ELA factors and brain controllability showed poor concordance across European and non-European participants (*r* = 0.073, *p* = 0.614), suggesting ancestry-specific effects. Among participants of European ancestry, F5 was positively associated with controllability in all ten networks (*β* = 0.042–0.088, *q* < .05, **Supplementary Fig. 11**), but these effects were attenuated or absent in non-European participants. By contrast, associations between ELA and behavioral outcomes, as well as brain controllability-behavior links, were broadly consistent across ancestry groups (**Supplementary Figs. 12–13**).

However, interaction analyses revealed significant moderation by ancestry in the associations between F5 and brain controllability (*q* < .05, **Supplementary Fig. 14, Supplementary Table 9**), particularly in the frontoparietal, default mode, and medial frontal networks. All interaction coefficients were positive, indicating that the association between F5 and brain controllability was stronger among participants of European ancestry. In other words, increases in socioeconomic disadvantage were more strongly related to alterations in controllability in European than in non-European participants. These findings underscore the importance of considering population-specific contexts in developmental neuroimaging studies.

### Mediation and Moderated Mediation Analyses

**Figure 4:**
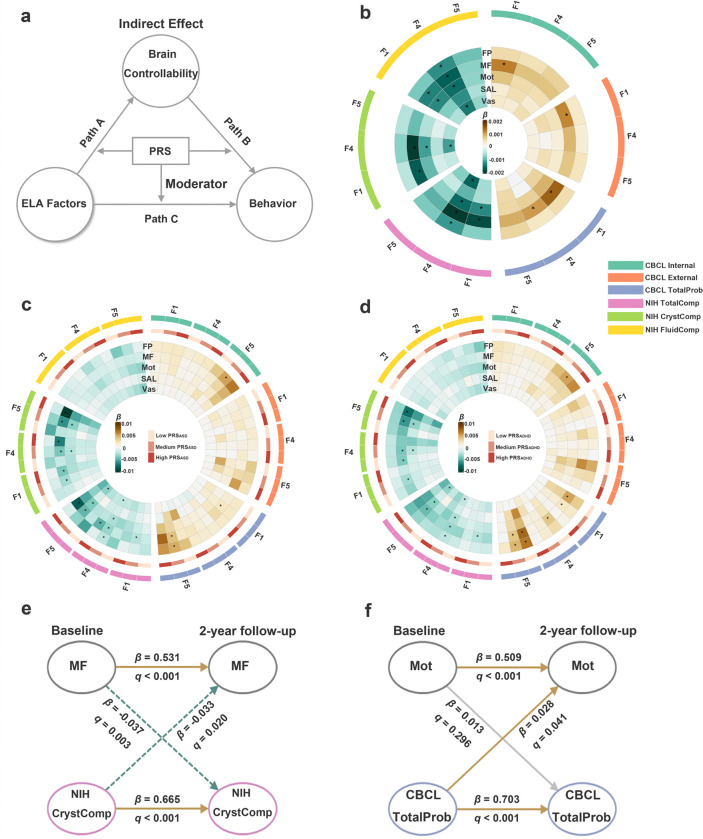
Mediation, Moderated Mediation, and Longitudinal Path Models Linking ELA, Brain Controllability, and Behavioral Outcomes. (A) The conceptual model of a fully moderated mediation framework, where ELA factors influence behavioral outcomes via brain controllability (indirect effect: Path A × Path B), with PRS moderating all three paths. (B) Mediation pathways from ELA factors to behavioral outcomes through brain controllability. (C, D) Moderated mediation pathways in individuals stratified by PRS_ASD_ (C) and PRS_ADHD_ (D) levels (low, medium, high). In (B-D), colors represent standardized regression coefficients (*β*), with significant indirect effects denoted by asterisks. (E) Cross-lagged panel model showing longitudinal bidirectional associations between controllability in the MF network and NIH crystallized cognition composite scores across baseline and 2-year follow-up. (F) CLPM results for the Mot network and CBCL total problem scores. Solid arrows indicate significant paths (*q* < .05); Yellow solid arrows indicate significant positive effects; green dashed arrows indicate significant negative effects; gray arrows denote non-significant paths. Abbreviations: F1: Paternal psychosocial adversity, F2: Family conflict/hostile environment, F3: Low caregiver and emotional support, F4: Caregiver psychopathology, F5: Socioeconomic disadvantages, FP, frontoparietal network, MF, medial frontal network, Mot, motor network, SAL, salience network, Vas, visual association network.

We tested a fully moderated mediation framework to examine whether brain controllability mediated the effects of ELA on cognitive and behavioral outcomes, and whether these indirect effects varied by genetic risk for neurodevelopmental disorders (Fig. 4A).

Mediation analyses revealed that brain controllability in specific brain networks partially mediated the associations between ELA factors and behavioral problems and cognitive performance (Fig. 4B and **Supplementary Table 10**). For instance, the medial frontal network controllability partially mediated the associations of F4 (caregiver psychopathology) and NIH cognitive scores, including the total composite (proportion mediated = 3.1%, indirect β = −0.002, 95% CI = −0.004 to −0.001), and crystallized composite (proportion mediated = 3.8%, indirect β = −0.002, 95% CI = −0.004 to −0.001). Medial frontal controllability also mediated the link between F4 and CBCL total problems (proportion mediated = 0.6%, indirect β = 0.001, 95% CI = 0.0002 to 0.002). Similarly, the frontoparietal network controllability partially mediated the association between F4 and total cognition score (proportion mediated = 4.8%, indirect β = −0.001, 95% CI = −0.002 to −0.0003).

To evaluate moderation by genetic risk, we stratified individuals based on PRS_ADHD_ or PRS_ASD_. In individuals with higher PRS_ADHD_ or PRS_ASD_, the indirect effects of F5 (socioeconomic disadvantage) on crystallized cognition via medial frontal network controllability were more pronounced compared to those with medium PRSs individuals. For example, among those with high PRS_ASD_, the moderated proportion mediated was 7.5% (indirect β = −0.012, 95% CI = −0.021 to −0.0006), compared to 3.7% in the medium PRS_ASD_ group (indirect β = −0.006, 95% CI = −0.011 to −0.0003). Similarly, in high PRS_ADHD_ individuals, the moderated proportion mediated was 3.9% (indirect β = −0.008, 95% CI = −0.017 to −0.0002) versus 3.6% in the medium PRS_ADHD_ group (indirect β = −0.006, 95% CI = −0.011 to −0.0002). These results suggest that genetic vulnerability amplifies the impact of ELA on cognition through altered brain controllability, potentially in a dose-dependent relationship. Additional significant statistically moderated mediations are reported in Fig. 4C/D, **Supplementary Table 11**).

### Longitudinal Associations Between Controllability and Behavioral Outcomes

We performed cross-lagged panel modeling (CLPM) to examine bidirectional relationships between brain controllability and behavioral outcomes over time. Analyses included 5,115 participants with 2-year follow-up data for behavioral problem and in 3,999 for crystallized cognition. All combinations of 10 brain networks and 4 outcomes (3 behavioral and 1 cognitive measures) were tested, but only a subset demonstrated significant longitudinal associations.

Autoregressive paths revealed strong within-domain stability over the 2-year interval, with all brain controllability and behavioral/cognitive variables showing significant self-prediction (e.g., medial frontal controllability: β = 0.531, *q* < .001; crystallized cognition: β = 0.665, *q* < .001; Fig. 4E, F; **Supplementary Tables 12, 13**).

Cross-domain associations were selective and often asymmetric (Fig. 4E, F; **Supplementary Tables 12, 13**). For example, higher medial frontal controllability at baseline predicted lower crystallized cognition at follow-up (β = −0.037; *q* = .003), and lower baseline cognition also predicted increased medial frontal controllability at follow-up (β = −0.033; *q* = .020), suggesting a bidirectional relationship between control dynamics and cognition development (Fig. 4E). In contrast, total behavioral problems at baseline predicted increased motor network controllability two years later (β = 0.028; *q* = .041), but not vice versa (β = 0.013; *q* = .296), indicating a unidirectional influence of behavior on brain development (Fig. 4F).

## Discussion

In this large, demographically diverse pediatric sample, we identified associations between specific ELA dimensions to distinct brain networks and, in turn, to cognitive and behavioral outcomes. Adversity related to caregiver psychopathology and paternal psychosocial disruption displayed the strongest associations with brain controllability in the medial frontal, frontoparietal, salience, and motor networks. These same networks were also the strongest mediators linking ELA to overall behavioral problems and crystallized cognition, suggesting that behavioral regulation and cognitive control circuits may be particularly sensitive to early psychosocial stress. Notably, the effects of ELA on behavior and cognition via controllability were moderated by polygenic risk for ADHD and ASD, highlighting a gene-environment interaction in shaping neurodevelopmental trajectories. Taken together, these findings provide compelling evidence for a structured neurodevelopmental cascade, from adversity to altered brain connectivity to behavioral impairment, refined further by inherited genetic risk. This work provides a systems-level framework for delineating how early adversity shapes neurodevelopmental pathways, offering new directions for predictive models and precision-oriented early interventions integrating neurobiology and genomics.

Historically, it has been difficult to disentangle the many early life experiences that drive neurodevelopment [72, 73]. A key strength of this study lies in the identification of five distinct and interpretable latent factors of ELA. This multidimensional approach incorporates the co-occurrence of ELA and supports growing evidence that ELA is not a unitary construct [72]. These results emphasize the importance of parsing the heterogeneity in ELA to characterize its downstream impact on the brain and behavior. For example, these latent factors exhibited unique associations with controllability and later behavior, which may have been occluded if ELA was treated as a unitary construct.

Although no prior studies have examined controllability in the context of ELA, our findings are broadly consistent with previous reports linking parental psychosocial adversity, psychopathology, and socioeconomic disadvantages to altered white matter integrity and functional connectivity in key regulatory networks, including the salience, medial frontal, frontoparietal, and cerebellar networks [74–78]. We found that paternal psychosocial adversity (F1) and caregiver psychopathology (F4) were associated with the controllability in the salience, frontoparietal, visual, and motor networks. These networks are involved in affect regulation and executive function [79–82]. These brain network alterations may reflect heightened allostatic demands and adaptive neurodevelopmental tuning in response to chronic caregiver-related stress. [15] Interestingly, socioeconomic disadvantage and neighborhood insecurity (F5) were associated with controllability in the salience and cerebellar networks, suggesting partially distinct neural pathways for deprivation-based versus relational adversity. These findings are consistent with prior work demonstrating that different types of ELA, such as threat versus deprivation, may exert dissociable effects on brain organization and function [83, 84]. The recurrent involvement of the salience network across multiple ELA dimensions in our findings underscores its potential role as a common transdiagnostic hub affected by early adversity. The well-established functions of salience network in detecting salient internal and external stimuli, mediating switching between major brain networks, and coordinating allostatic responses may account for its vulnerability to early environmental stressors [85]. From a neurodevelopmental perspective, repeated stress exposure may recalibrate the controllability of salience network, potentially enhancing their influence over dynamic brain states in an effort to maintain functional homeostasis. This hypothesis is supported by emerging evidence that early life adversity may accelerate the maturation or functional coupling of salience-related circuits [73, 86–89]. Consistent with this view, a study reported that young adults with a history of childhood trauma exhibited increased network switching rates, reflecting reduced temporal stability of functional connectivity, especially within the frontoparietal and cingulo-opercular networks [90]. These results suggest that early adversity may lead to more dysregulated network dynamics, potentially increasing the allostatic load required to maintain cognitive and emotional homeostasis.

Notably, the ancestry-specific moderation was observed for the association between the socioeconomic adversity (F5) and brain controllability. Participants of European ancestry showed steeper positive slopes between F5 and controllability in the medial frontal, frontoparietal, default mode, and salience networks. One possible interpretation is that, in relatively resource-advantaged contexts, socioeconomic adversity may represent a greater deviation from normative expectations, thereby exerting a stronger physiological and neurodevelopmental impact. This interpretation aligns with prior research suggesting that the magnitude of brain-environment associations depend not only on the severity of exposure but also on the distributional range and contextual meaning of socioeconomic risk [75].

In several brain networks, higher controllability associated with greater behavioral problems and worse cognitive abilities. Controllability reflects the theoretical ability of a network to drive the brain into difficult-to-reach states [31]. Thus, higher controllability may indicate greater neural instability or inefficient regulation, especially under higher allostatic loads. Further, controllability typically increases in adolescents [32]. Greater controllability in the context of ELA may also reflect accelerated development [9, 91], which is commonly observed in response to ELAs. These associations emphasize that higher controllability should not be interpreted as uniformly advantageous or disadvantageous. Together, the findings highlight the importance of contextualizing controllability and other neuroimaging measures within developmental histories when evaluating their functional significance.

Mediation analyses revealed that brain controllability served as a partial mechanism linking ELA to both cognitive deficits and behavioral problems. These indirect effects were most prominent through the medial frontal and frontoparietal networks, regions central to cognitive control and affective processing [92–94]. Conceptually, these results are consistent with a prior network control theory study showing that lower control energy needed to engage frontoparietal networks associates with better executive performance and partly mediates age-related gains in cognition [95]. Further, our results extend previous work by demonstrating that controllability can also act as a partial mediator of adversity-related outcomes. Notably, these mediation effects were further moderated by polygenic risk for ADHD and ASD. The inclusion of these mediation and moderated mediation models is a strength and nuances the understanding of individual variability in neurodevelopment. Overall, they support a “double-hit” model, where ELA and genetic vulnerability jointly influence neurodevelopment through alterations in brain controllability [96, 97].

The longitudinal findings indicate that bidirectional associations between brain controllability and behavior exist, indicating that cognitive abilities and controllability mutually shape each other during adolescence. This dynamic aligns with the sensorimotor-association axis of cortical maturation, wherein higher order association regions involved in cognitive functions undergo prolonged development and plasticity [98]. In contrast, the unidirectional effect of early behavioral problems on later motor network controllability indicates that dysregulation may shape the motor network development. This finding is partially supported by structural imaging studies showing that persistent externalizing behaviors predict altered subcortical maturation and disrupted prefrontal-limbic coupling [99]. However, cross-lagged panel models account for temporal stability using autoregressive paths and do not distinguish between-person differences and within-person fluctuations. These models reflect group averages, rather than casual individual differences [71].

This study has several limitations. First, the ELA factors were based on caregiver and child reports of past experiences, which could be influenced by recall or reporting bias. Second, PRS analyses were limited to participants of European ancestry; findings may not extend to more diverse populations. Finally, our models examined only linear associations and did not account for possible nonlinear effects.

Using data from the ABCD study, we revealed that ELA dimension-specific influences on brain controllability, which in turn affected behavioral outcomes in children. Genetic vulnerability further moderated these relationships, underscoring the interplay between genes and environment in shaping neurodevelopment. Ultimately, identifying individual-level brain markers (e.g., controllability) could help improve early risk detection and guide personalized intervention strategies.

## Supplementary Material

Supplementary Files

This is a list of supplementary files associated with this preprint. Click to download.

• Supplementarymaterials.docx

## Figures and Tables

**Table 1:** Demographic Characteristics of Participants Included in the Study.

Demographic Characteristics	No. (%/SD)
Age, mean (SD), y	9.47 (0.51)
Sex	
Male	4141 (52.0)
Female	3 829
Pubertal Development Scale Score	−0.008 (0.97)
Handedness	
Right-handed	6365 (79.9)
Left-handed	560 (7.0)
Mix-handed	1045 (13.1)
Race^a^	
Asian	136 (1.7)
Black	1193 (15.0)
Hispanic	1602 (20.1)
White	4228 (53.0)
Other^b^	811 (10.2)
Child Behavior Checklist (CBCL)^c^	
CBCL Internal	48.6 (10.6)
CBCL External	45.7 (10.2)
CBCL Totalprob	46.0 (11.2)
NIH Toolbox Cognition^d^	
NIH FluidComp	45.9 (10.8)
NIH CrystComp	51.4 (11.0)
NIH TotalComp	48.1 (10.8)

a Race was reported by each participant’s caregiver.

b Other includes American Indian or Alaska Native, Native Hawaiian, and Pacific Islander participants.

c CBCL scores are derived from caregiver report using empirically based syndrome scales: internalizing (CBCL Internal), externalizing (CBCL External), and total problems (CBCL Totalprob).

d Cognitive scores were obtained from the NIH Toolbox Cognition Battery, including fluid intelligence (NIH FluidComp), crystallized intelligence (NIH CrystComp), and total composite score (NIH TotalComp).

## Data Availability

Data used in the preparation of this article were obtained from the Adolescent Brain Cognitive Development Study^®^ (https://abcdstudy.org), held in the NIMH Data Archive (NDA). Only researchers with an approved NDA Data Use Certification (DUC) may obtain ABCD Study data.

## Code availability

We made use of publicly available software and tools. The publicly available tools used in our analyses are described in Methods. The core codes are available at https://github.com/haigui0913/ELA_Controllability_Behavior_ABCD.
